# Factors associated with high exclusive breastfeeding rates among preterm infants under 34 weeks of gestation in Da Nang, Vietnam: A retrospective cohort study

**DOI:** 10.7189/jogh.13.04121

**Published:** 2023-11-09

**Authors:** Hoang Thi Tran, Hanh My Luu, Thao Dieu Le, Nga Thi Quynh Pham, Howard L Sobel, JCS Murray

**Affiliations:** 1 Neonatal Unit, Da Nang Hospital for Women and Children, Da Nang, Vietnam; 2 Department of Paediatrics, School of Medicine and Pharmacy, Da Nang University, Vietnam; 3 World Health Organization Representative Office in Viet Nam, Ha Noi, Vietnam; 4 World Health Organization Western Pacific Regional Office, United Nations Avenue, Manila, Philippines

## Abstract

**Background:**

Preterm infants have higher mortality than full-term infants. While breastfeeding dramatically reduces preterm death, it is limited by biological and practice barriers, particularly for babies born before 34 weeks gestational age. Da Nang Hospital for Women and Children developed a quality improvement approach to improve breastfeeding of preterm infants by strengthening feeding support, non-separation, and kangaroo mother care (KMC).

**Methods:**

To determine breastfeeding outcomes following discharge and explore factors associated with improved feeding, mothers of infants under 34 weeks gestational age born October 2021 to March 2022 and discharged alive were interviewed at six months and their medical records were reviewed.

**Results:**

Out of 104 preterm infants included, all were exclusively breastfed at discharge and one month, 86.5% at three months, and 63.5% at six months; 47.1% received immediate skin-to-skin contact, 31.7% immediate and continuous KMC, and the remaining 68.3% continuous KMC beginning at a median of three days. Exclusive breastfeeding at six months was associated with the mother antenatally seeking breastfeeding information (odds ratio (OR) = 14.5; 95% confidence interval (CI) = 1.2-173.6), avoiding bottle-feeding at home (OR = 7.7; 95% CI = 1.7-33.7) and reduced with each day delay between birth and full breastfeeding (OR = 0.8; 95% CI = 0.6-0.9).

**Conclusions:**

Hospital environments that limit mother-baby separations and feeding delays, including rooming-in of mothers and infants, KMC, and breastfeeding support from birth, enabled 100% of preterm infants born before 34 weeks gestational age to breastfeed exclusively with continued rates higher than previously reported. Addressing antenatal and post-natal factors limiting practice can further improve longer-term breastfeeding outcomes. The approach can be adapted to achieve high exclusive breastfeeding rates, regardless of gestational age.

Early and exclusive breastfeeding (EBF) for six months of life reduces child mortality and morbidity and improves growth and development. Early breastfeeding specifically prevents neonatal hypoglycaemia, sepsis, diarrhoea, and pneumonia [1], while breastfeeding overall is associated with reduced childhood metabolic disorders, leukaemia, type 2 diabetes, obesity and allergies, and increased intelligence quotient (IQ) [2]. Preterm infants (born before 37 weeks of gestational age) are at particularly high risk of mortality (an estimated 17% of global under-five child deaths in 2019) and long-term adverse outcomes. Early and EBF improves preterm survival and reduces the risk of necrotising enterocolitis, retinopathy of prematurity, and bronchopulmonary dysplasia. Applying KMC reduces preterm mortality by 32%, with immediate and continuous KMC after birth being more effective than if it is delayed until after stabilisation [3].

In Vietnam, early breastfeeding initiation fell nationally between 2011 and 2020, from 39.7 to 23.5% with bottle-feeding rates rising from 38.7 to 54.3%. Only 46% of babies were exclusively breastfed for the first 6 months of life in 2020, although this was an improvement from 17% in 2011 [4]. Low breastfeeding rates are noted despite laws and policies to protect, promote, and support breastfeeding [5], and facility-based initiatives, including Early Essential Newborn Care (EENC) and KMC [6].

An estimated 6.5% of babies were born preterm in Vietnam in 2014, with higher rates observed in hospitals [7,8]. Preterm infants are breastfed less than term infants both in Vietnam and globally [6,9]. Numerous factors may limit breastfeeding. For example, preterm infants have reduced ability to suckle and breastfeed, with those born less than 34 weeks gestational age having undeveloped suck and swallow reflexes. Some newborns have complications after birth, such as respiratory distress, that require intensive care. Mothers can have rare or urgent maternal medical conditions requiring separation of the baby. Practice protocols often routinely separate stable preterm infants at birth for observation, and thus do not allow application of immediate skin-to-skin contact (SSC) [6,10]. Long-term, preterm infants often have poorer growth compared to full-term newborns, which may lead mothers to choose formula, further contributing to mortality and morbidity [11].

The Da Nang Hospital for Women and Children (DNHWC) began implementing EENC interventions in 2014. Before introduction, the EBF rate among full-term babies in the hospital was 27%, rising to more than 95% in 2022 [12]. To improve breastfeeding practices of preterm infants, a quality assurance approach was developed by adapting EENC to limit mother-baby separations, strengthen feeding support, and apply early continuous KMC. To assess its progress, we conducted a retrospective cohort study on breastfeeding practice outcomes and factors affecting breastfeeding among preterm infants graduating from the neonatal unit (NU).

## METHODS

### Setting

The DNHWC is a regional referral hospital for obstetrics, gynaecology, and paediatrics, serving three provinces in central Vietnam with a population of four million. The hospital has approximately 15 000 annual births, 61% of which are conducted by caesarean section [12]. More than 30% of admissions are women with high-risk pregnancies and children with high-acuity illnesses [13]. There are 1200 beds including 22 standard delivery beds and four operative delivery beds. The NU has 120 beds, including 30 level III beds with mechanical ventilator support and care for extremely low birthweight newborns, 50 beds for KMC, and 40 beds for high dependency care.

### Study population and procedures

We conducted this retrospective cohort study from 1 February 2022 to 30 September 2022 on all infants under 34 weeks of gestation born between 1 October 2021 and 31 March 2022, alive at discharge from the NU at DNHWC. We surveyed the mothers of discharged preterm infants once at six months after birth using standardised questionnaires to collect retrospective data on infant weight (from home health records), breastfeeding status at one, three, and six months of age, feeding practices, information and counselling received, work practices, and socioeconomic variables. Only the first-born infant of mothers with multiple births was selected for data collection. We trained three doctors in administering the questionnaire to ensure inter-observer reliability. These doctors called sampled mothers using telephone numbers from the hospital database and conducted interviews after securing verbal consent from each participant. Each doctor interviewed around 35 mothers. We used medical records to obtain general characteristics (demographics, maternal history, birth weight, and gestational age), in-patient progress in the NU (respiratory support and enteral feeding received, timing of the first feed, and timing of KMC), discharge status of the newborn (weight, breastfeeding status, postconceptional age at discharge, and length of hospital stay), and re-admissions. We used the study findings to frame focus-group discussions and ward observations with staff in maternity and the NU, to generate qualitative data on reasons for practice gaps and barriers.

### NU preterm management protocols

All stable newborns at DNHWC receive immediate SSC with the mother in the delivery or operating room. Preterm infants above 34 weeks of gestation and 1800 grams continue SSC as KMC in the postnatal wards and those below 34 weeks of gestation, under 1800 grams, or requiring respiratory support in the NU.

NU staff counsel and support mothers and families to provide KMC for all preterm infants as soon as possible after admission until discharge or at 40 weeks of gestation. Preterm infants are given exclusively breastmilk directly from breast, cup or spoon or via gastric tube combined with parenteral nutrition depending on gestational age, birth weight, and stability. If breastmilk is not available, they receive pasteurized donor breastmilk from the milk bank. From 32 weeks of gestation, preterm infants are supported to practice breastfeeding. Direct breastfeeding is delayed if respiratory support with a high oxygen requirement is required.

The hospital issues, periodically assesses, and enforces numerous policies on EENC and breastfeeding. Infant formula use is forbidden. All staff in maternal and newborn units are trained and certified using hospital breastfeeding counselling and EENC competency standards, while all staff involved in care of preterm babies are trained and certified in KMC. Pregnant women are counselled in antenatal clinics and the pre-birth suite on the importance of breastfeeding, basic breastfeeding practices, feeding cues for breastfeeding readiness, and the advantages of SSC. All stable babies and babies who begin breathing after resuscitation receive core EENC practices, including immediate and uninterrupted SSC for at least 90 minutes, delayed cord clamping, and early and EBF (including support for correct breastfeeding positioning and attachment). All preterm infants are roomed-in and mothers supported to provide immediate and continuous KMC as soon as possible. Practice observations and maternal exit interviews using standard checklists are periodically conducted to review EENC implementation and breastfeeding practices to inform quality improvement activities [6]. Discharge planning including a detailed follow-up schedule and counselling on danger signs for immediate care-seeking are given to all mothers.

### Variable definitions

Maternal and newborn variable definitions are presented in **Box 1**.

Box 1 **Maternal and newborn variable definitions.**

**Maternal variables**
− Age (years);
− Education (high school diploma level or below, completed two-year post-school diploma, completed college degree, completed university degree);
− Employment status (business/self-employed, farmer/blue-collar worker, white-collar worker, homemaker, student, others),
− Place of residence (Da Nang city or other);
− Family income (less than, greater, or equal to 20 million Viet Nam Dong monthly, the low-income threshold as defined by the General Statistics Office of Vietnam, 2021);
− Number of children (one, two, three or more);
− Place of antenatal care (hospitals, private clinics, or both);
− Maternal medical problems during pregnancy: Clinical diagnosis at any time during pregnancy of gestational hypertension, gestational diabetes, thyroid disease, or other medical problems; Antenatal breastfeeding consultation: At least one antenatal breastfeeding support visit at a public, private or other antenatal care site;
− Seeking information on breastfeeding before childbirth: The mother sought information about breastfeeding during her pregnancy from any source (e.g. antenatal provider, print or other media, family, community providers);
− Mode of delivery: Vaginal or caesarean section;
− Place of delivery: Inborn (DNHWC) and outborn (other hospitals);
− Exposed to formula milk advertising: The mother reported seeing or receiving any infant formula promotion (e.g. print or electronic media, gifts, samples) within six-months after birth;
− Went back to work within six months after birth: The mother reported any paid employment, full or part-time, within six-months of birth, regardless of employment type or duration.

**Newborn variables**
− Immediate SSC: The newborn was dried immediately after birth and then placed into direct SSC on the mother’s chest or abdomen without separation;
− Immediate and continuous KMC: Immediate SSC performed with no separation until KMC, which was applied for at least 20 hours in each 24-hour period, with no separation exceeding 30 minutes, until discharge or 40 weeks;
− Newborn respiratory support: Any support including oxygen canula, continuous positive airway pressure (CPAP), non-invasive or invasive ventilation;
− Time to achieve full feeding with mother’s breastmilk: The time in days between birth and feeding with only mother’s own breastmilk, without use of donor breastmilk. Feeds could be given by gastric tube, cup, or direct breastfeeding;
− Corrected age at starting direct breastfeeding: The corrected age in days at the time the preterm infant began direct breastfeeding;
− Corrected age at full direct breastfeeding: The corrected age in days at the time the preterm infant was fully fed by direct breastfeeding;
− Breastfeeding at discharge, one, three and six months: Feeding practices were categorised as EBF (fed only breastmilk except for medication), predominant breastfeeding (fed mostly breastmilk with water and water-based fluids or formula in smaller amounts), mixed feeding (fed breastmilk and formula, water or water-based fluids in comparable amounts), predominant formula feeding (fed mostly formula), and exclusive formula feeding;
− Weight for Age: The Fenton World Health Organization (WHO) growth chart was used at birth until 50 weeks of corrected age. Small for gestational age was under the 10^th^ weight percentile [14]. The WHO growth chart from birth to six months was used after 50 weeks of corrected age [15]. Small for gestational age was defined as weight under the 3^rd^ percentile.

### Statistical analyses

We used SPSS, version 26.0 (IBM Corp, New York, USA) for statistical calculations. We presented normally distributed continuous variables as means and standard deviations, skewed continuous variables as medians and interquartile ranges (IQR), and categorical variables as counts and percentages. We compared the variables between the two groups using the Student’s independent samples t-test for continuous variables and the χ^2^ test for categorical variables. We assessed associations between EBF at six months and maternal and newborn factors associated with breastfeeding practice using logistic regression and presented the results as odds ratios and 95% confidence intervals (CIs). We considered a *P*-value <0.05 as statistically significant.

### Ethical approval

The study was conducted according to the Declaration of Helsinki, approved by the Ethics Committee of Da Nang Hospital for Women and Children (Protocol code 2022.12.15); and waived for ethical review by WHO Ethical Review Committee. We obtained informed verbal consent prior to maternal post-discharge interviews and did not use any personal identifiers.

## RESULTS

**Figure 1:**
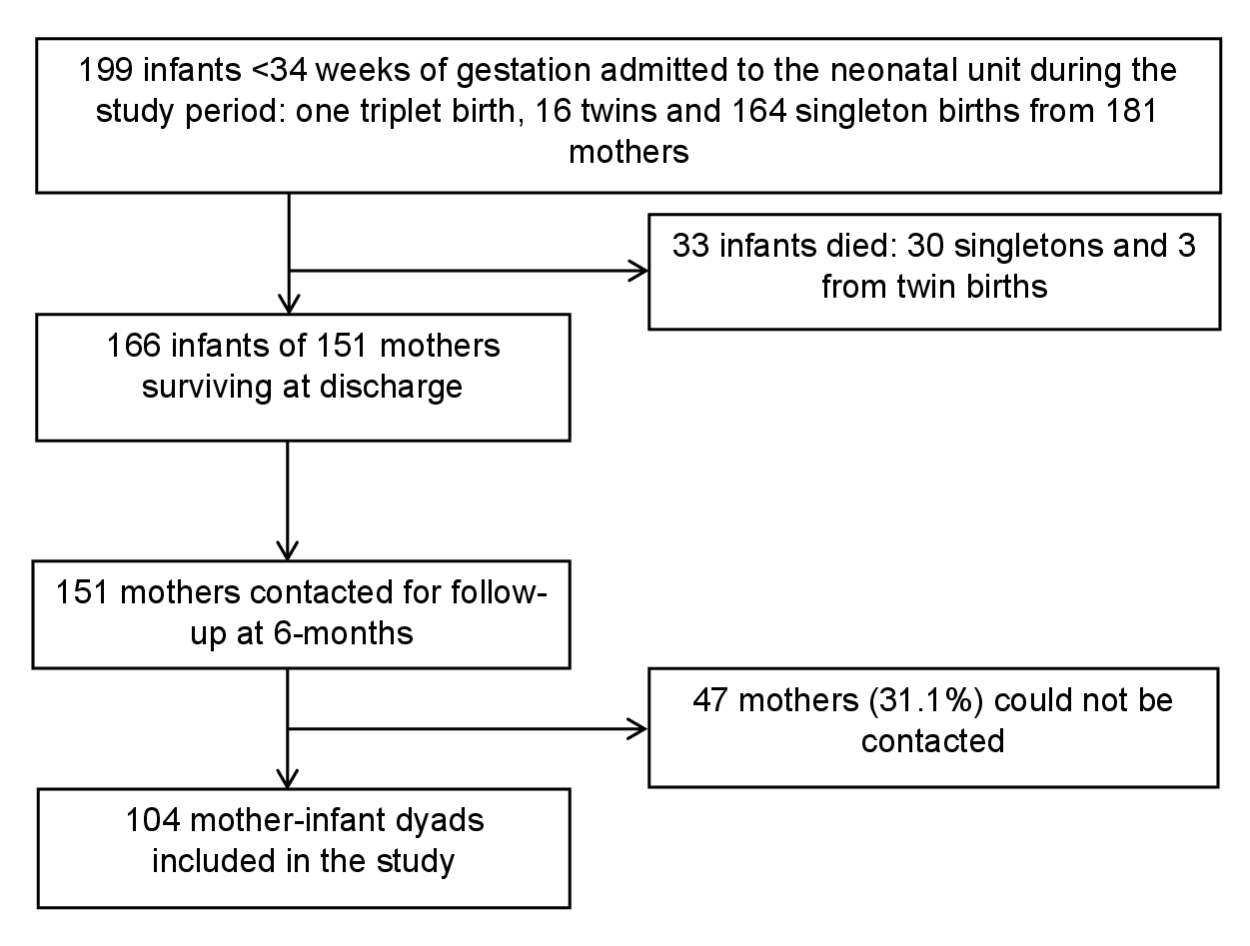
Flow chart summarising the sampling of mother-infant dyads included in the preterm follow-up study, Da Nang Hospital for Women and Children, October 2021 to March 2022.

During the study period, 199 infants <34 weeks of gestation were admitted to the NU, including one triplet birth, 16 twins, and 164 singletons born by 181 mothers. Thirty-three (16.6%) of included preterm infants died and 166 (83.4%) survived. Three babies from twin births and 30 singleton births resulted in preterm deaths, leading to the exclusion of 30 mothers from the sample, leaving a total of 151 mothers. Forty-seven of the remaining mothers could not be contacted for an interview, resulting in a final sample of 104 (68.9%) mother-infant dyads (**Figure 1**).

Eighty-one (77.9%) of the included mothers were aged between 20 and 35 years; most had business or white-collar jobs (n = 64 (61.5%)), higher education (71, 68.3%), resided in another province (n = 66 (63.5%)), gave birth to their first child (n = 57 (54.8%)), and had C-section birth (n = 56 (53.8%)). Thirty-three (31.7%) sought antenatal care from private clinics, 36 (34.6%) from hospital clinics and 35 (36.7%) from both. Twenty-seven (26.0%) mothers had medical problems during pregnancy (**Table 1**).

**Table 1:** Characteristics of mothers of preterm infants admitted to the neonatal unit, Da Nang Hospital for Women and Children, October 2021 to March 2022*

Maternal age in years, mean (SD)	30.2 (5.2)
<20	3 (1.9)
20-35	81 (77.9)
>35	20 (19.2)
**Occupation**	
Business	20 (19.2)
Farmer/Worker	14 (13.5)
White collar jobs	44 (42.3)
Housewife	25 (24.0)
Student	1 (1.0)
**Education**	
High school diploma or lower	33 (31.7)
Diploma, college	34 (32.7)
University and higher	37 (35.6)
Place of residence	
Da Nang	38 (36.5)
Other provinces	66 (63.5)
	
**Family income per month ≥20 million VND**	19 (18.0)
**Number of children**	
1	57 (54.8)
2	31 (29.8)
3 or more	16 (15.4)
**Place of antenatal care**	
Private clinics	33 (31.7)
Hospital clinics	36 (34.6)
Both	35 (33.7)
**Medical problems during pregnancy**	
Gestational diabetes	9 (8.7)
Gestational hypertension	10 (9.7)
Thyroid disease	4 (3.8)
Others	4 (3.8)
None	77 (74.0)
**Prenatal breastfeeding support**	
Antenatal breastfeeding consultation	77 (74.0)
Sought information on breastfeeding before childbirth	96 (92.3)
**Mode of delivery**	
Vaginal	48 (46.2)
Caesarean	56 (53.8)
**Place of delivery**	
Inborn	95 (91.3)
Outborn	9 (8.7)
**Post-discharge practices**	
Exposed to formula milk advertisements	29 (27.8)
Went back to work within six months of birth	26 (25)

VND – Vietnamese Dong

*Values presented as n (%) unless specified otherwise.

Of the 104 included preterm infants, 95 (91.3%) were born at DNHWC and 10 (9.6%) delivered at less than 28 weeks of gestation. The mean gestational age at birth was 30.6 weeks and mean birth weight 1548.6 grams, meaning 92 (88.5%) infants had appropriate weight for gestational age. Seventeen (16.3%) required no respiratory support, 80 (76.9%) required CPAP, six (5.8%) required mechanical ventilation, and one (1%) required oxygen. The median duration of respiratory support when received was six days (**Table 2**).

**Table 2:** Characteristics of preterm infants admitted to the neonatal unit, Da Nang Hospital for Women and Children, October 2021 to March 2022*

Gestational age in weeks, mean (SD)	30.6 (2.2)
<28 weeks, mean (SD)	10 (9.6)
28+0-31+6 weeks, mean (SD)	49 (47.1)
32+0-33+6 weeks, mean (SD)	45 (43.3)
**Gender**	
Male	59 (56.7)
**Birthweight in grams, mean (SD)**	1548.6 (466.4)
<1000, mean (SD)	15 (14.4)
1000-1499, mean (SD)	42 (40.4)
≥1500, mean (SD)	47 (45.2)
**Weight-for-age at birth**	
Appropriate for gestational age	92 (88.5)
Small for gestational age	7 (6.7)
Large for gestational age	5 (4.8)
**Respiratory support**	
No respiratory support	17 (16.3)
Mechanical ventilation	6 (5.8)
CPAP	80 (76.9)
Oxygen	1 (1)
Duration of respiratory support in days, median (IQR)	6 (1-22)
**Essential newborn care**	
Skin-to-skin contact at birth	49 (47.1)
Immediate and continuous KMC	32 (31.7)
KMC during hospital stay	104 (100)
Time to begin KMC in days, median (IQR)	3 (1-7)
**Feeding practices in hospital**	
First feed – mother’s breastmilk	43 (41.3)
First feed – donor breastmilk	61 (58.7)
First time expressing breastmilk ≤6 hours	33 (31.7)
Time to start mother’s breastmilk in days, median (IQR)	2.0 (1-3)
Time to full feeding with mother’s breastmilk in days, median (IQR)	4 (3-6.8)
Corrected gestational age at starting cup/spoon-feeding in weeks, mean (SD)	33.5 (1.5)	
Corrected gestational age at starting direct breastfeeding from the mother in weeks, mean (SD)	34.2 (1.6)
Corrected gestational age at full direct breastfeeding in weeks, mean (SD)	35.0 (1.9)
**Post-discharge feeding practices in the first six months**	
**Used feeding bottle**	75 (72.1)
Mother expressed breastmilk into bottles	70 (67.3)
Rehospitalisation	
None	63 (60.6)
1 time	23 (22.1)
≥2 times	18 (17.3)

SD – standard deviation, IQR – interquartile range, KMC – Kangaroo Mother Care, CPAP – continuous positive airway pressure

*Values presented as n (%) unless specified otherwise.

Forty-nine (47.1%) preterm infants received SSC with the mother immediately after birth, and all received KMC, with 33 (31.7%) receiving immediate and continuous KMC and 71 (68.3%) beginning KMC at a median of three (IQR = 1-7) days after birth. All preterm infants received breastmilk as the first feed, of whom 43 (41.3%) received their mother’s own breastmilk and the remaining 61 (58.7%) donor breastmilk. Thirty-three (31.7%) mothers expressed breastmilk initially within six hours of birth, with full feeding with mother’s breastmilk beginning at a median of four days. The mean gestational age at which direct breastfeeding from the mother began was 34.2 weeks, with full direct breastfeeding beginning at 35.0 weeks. The mean age at discharge was 35.6 weeks (**Table 2**).

All 104 infants were exclusively breastfed at discharge and at one month, 90 (86.5%) at three months, and 66 (63.5%) at six months old. The proportion of small for corrected age declined from 45 (43.2%), 39 (37.5%), 36 (34.6%), and 18 (17.3%) at discharge, one, three and six months, respectively. Around 41% of preterm infants had at least one rehospitalisation (**Table 3**).

**Table 3:** Feeding practices and growth data from discharge to six months, preterm infants admitted to the neonatal unit, Da Nang Hospital for Women and Children, October 2021 to March 2022*

Discharge	
Exclusive breastfeeding	104 (100.0)
Small for corrected age	45 (43.2)
Appropriate for corrected age	58 (55.8)
Large for corrected age	1 (1.0)
Corrected age at discharge in weeks, mean (SD)	35.6 (2.2)
Weight at discharge in grams, mean (SD)	2038.9 (438.5)
**One month old**	
Exclusive breastfeeding	104 (100.0)
Small for corrected age	39 (37.5)
Appropriate for corrected age	64 (61.5)
Large for corrected age	1 (1.0)
**Three months old**	
Exclusive breastfeeding	90 (86.5)
Predominant breastfeeding	4 (3.8)
Mixed feeding	1 (1.0)
Predominant formula	7 (6.6)
Exclusive formula feeding	2 (1.9)
Small for corrected age	36 (34.6)
Appropriate for corrected age	62 (59.6)
Large for corrected age	6 (5.8)
**Six months old**	
Exclusive breastfeeding	66 (63.5)
Predominant breastfeeding	7 (6.7)
Mixed feeding	13 (12.5)
Predominant formula feeding	10 (9.6)
Exclusive formula feeding	8 (7.7)
Appropriate for corrected age	82 (78.8)
Small for corrected age	18 (17.3)
Large for corrected age	4 (3.8)

SD – standard deviation

*Values presented as n (%) unless specified otherwise.

In multi-variable analysis, EBF at six months of age was significantly associated with the mother seeking information about breastfeeding before childbirth (OR = 14.5; 95% CI = 1.2-173.6, *P* = 0.03) and not expressing breastmilk into bottles for feeding after discharge (OR = 7.7; 95% CI = 1.7-33.7, *P* = 0.008). EBF was significantly less likely with each day delay between birth and full breastfeeding (OR = 0.8; 95% CI = 0.6-0.9, *P* = 0.004) (**Table 4**). We noted a non-significant association between exclusive breastfeeding at six months of age and attending antenatal clinics at public hospitals (OR = 3.2; 95% CI = 0.9-11.2, *P* = 0.06) and having no medical problems in pregnancy (OR = 0.2; 95% CI = 0.04-1.0, *P* = 0.05).

**Table 4:** Multi-variable analysis of factors associated with exclusive breastfeeding at six months among preterm infants admitted to the neonatal unit, Da Nang Hospital for Women and Children, October 2021 to March 2022

Variable	Exclusive breastfeeding (n = 66), n (%)	Non-exclusive breastfeeding (n = 38), n (%)	Univariable analysis, OR (95% CI)	*P*-value	Multivariable analysis, adjusted OR (95% CI)	*P*-value
**Maternal**						
Maternal education – university or higher	25 (33.7)	12 (31.5)	1.3 (0.6-3.1)	0.5	3.4 (0.9-12.9)	0.07
Maternal age ≥20	64 (96.9)	37 (97.3)	0.9 (0.8-9.9)	0.01	0.4 (0.01-14.8)	0.6
Family income >20 million VND/month	15 (22.7)	4 (10.5)	2.5 (0.8-8.2)	0.1	2.7 (0.6-12.3)	0.1
Antenatal clinics at public hospitals	49 (74.2)	22 (57.8)	2.1 (0.9-4.9)	0.06	3.2 (0.9-11.2)	0.06
Antenatal breastfeeding counselling received	51 (77.3)	26 (68.4)	1.6 (0.6-3.8)	0.3	1.7 (0.4-6.6)	0.5
**Sought information about breastfeeding before childbirth**	64 (96.9)	32 (84.2)	6.0 (1.1-41.4)	0.02	14.5 (1.2-173.6)	0.03
No medical problems in pregnancy	47 (71.2)	30 (78.9)	0.7 (0.3-1.7)	0.4	0.2 (0.04-1.0)	0.05
Born at DNHWC	59 (89.3)	36 (94.7)	0.5 (0.9-2.4)	0.4	0.4 (0.05-4.0)	0.5
Normal delivery	31 (46.9)	17 (44.7)	1.1 (0.5-2.4)	0.8	0.8 (0.2-2.8)	0.3
Not exposed to formula milk advertisement	47 (71.2)	28 (73.6)	0.9 (0.4-2.2)	0.8	0.9 (0.2-3.5)	0.8
Mother started working after six months postnatal	48 (72.7)	30 (78.9)	0.7 (0.3-1.8)	0.5	0.7 (0.19-3.0)	0.7
**Infant**						
From second child	33 (50.0)	14 (36.8)	1.8 (0.8-4.0)	0.2	0.9 (0.3-2.8)	0.9
GA>32 weeks	29 (43.9)	16 (42.1)	1.1 (0.5-2.4)	0.9	1.1 (0.3-4.5)	0.9
Skin to skin after birth	31 (46.9)	18 (47.3)	1.0 (0.4-2.2)	1.0	0.3 (0.06-1.5)	0.2
Immediate and continuous kangaroo mother care	23 (34.8)	10 (26.3)	1.5 (0.6-3.6)	0.4	2.2 (0.4-11.6)	0.4
No respiratory support	11 (16.6)	6 (15.7)	1.1 (0.4-3.2)	0.9	0.5 (0.06 -3.9)	0.5
Not small for corrected age at one month	42 (63.6)	22 (57.8)	1.3 (0.6-2.9)	0.6	1.2 (0.3-4.5)	0.8
Not small for corrected age at three months	43 (65.1)	23 (60.5)	1.2 (0.5-2.8)	0.6	1.1 (0.3-4.5)	0.9
No rehospitalisation	41 (62.1)	22 (57.8)	1.2 (0.5-2.7)	0.7	1.1 (0.3 -3.2)	0.9
**Feeding practices**						
Mother expressed milk before six hours	24 (36.3)	9 (23.6)	1.8 (0.7-4.5)	0.2	1.7 (0.4-7.5)	0.5
First feeding of breastmilk	29 (43.9)	14 (36.8)	1.3 (0.6-3.1)	0.5	0.8 (0.2-3.0)	0.8
**Time to achieve full feeding with mother’s breastmilk in days, mean (SD)**	4.4 (3.3)	8.0 (8.3)	0.9 (0.8-1.0)	0.006	0.8 (0.6-0.9)	0.004
Corrected gestational age at full direct breastfeeding, mean (SD)	35.0 (1.9)	35.0 (1.9)	1.0 (0.8-1.2)	0.9	1.1 (0.8-1.5)	0.6
**No expressed milk into bottles after discharge**	26 (39.3)	7 (18.4)	3.0 (1.1-7.7)	0.02	7.7 (1.7-33.7)	0.007

SD – standard deviation, VND - Vietnamese đồng, DNHWC – Da Nang Hospital for Women and Children, GA – gestational age, OR – odds ratio, CI – confidence interval

## DISCUSSION

All 104 preterm infants under 34 weeks gestational age at birth who were discharged from the NU at DNHWC were exclusively breastfed at discharge and one month, 90 (86.5%) at three months, and 66 (63.5%) at six months. All received either immediate and continuous KMC (31.7%) or continuous KMC beginning at a median of three days after birth (68.3%). EBF was significantly associated with the mother seeking information on breastfeeding before childbirth and not expressing breastmilk into bottles for feeding at home, and significantly less likely with each day delay between birth and full feeding with mother’s breastmilk.

EBF rates for preterm infants at six months in our study were higher than those for all children in Vietnam and globally, estimated to be 49% in 2019 and 46% in 2020 [4,16]. These rates were higher than those reported for preterm infants in other hospital-based study populations in Shanghai, China (47.8% at discharge and 10.4% at six months) [17], Denmark (70.4% at hospital discharge and 35.4% at six months) [18] and Brazil (99.0% at hospital discharge and 8.0% at six months) [19]. Moreover, preterm infants in our study were more premature than in the Chinese study population (of whom 81.6% were 35-36 weeks of gestation) and those in Denmark and Brazil (for whom the median gestational age was 34 weeks).

Preterm infants initiated direct breastfeeding at a mean gestational age of 34.2 (standard deviation (SD) = 1.6) weeks and full direct breastfeeding at 35.0 (SD = 1.9) weeks, compared to previously reported rates of 34.4 (SD = 1.8) weeks and 36.7 (SD = 1.2) weeks [20]. The negative association noted in this study between EBF at six months and each day of delay between birth and full breastmilk feeding is consistent with previous studies [9,20]. Preterm infants are known to have reduced capacity to coordinate sucking, swallowing and breathing due to immature neural development and associated respiratory problems, with full breastfeeding usually possible around 34 weeks of gestation [21]. Earlier breastfeeding can be achieved with earlier introduction of oral feeding, early SSC, and KMC, all of which were applied for our study population [22]. Immediate SSC, received by half (47.1%) and KMC received by all preterm infants, were both previously found to be strongly associated with improved EBF rates both at hospital discharge and up to six months after birth [23].

The positive association between EBF and seeking information in the antenatal period is consistent with findings that positive attitudes towards breastfeeding and higher mean breastfeeding knowledge test scores are associated with improved EBF rates [24,25]. Improved attitudes and knowledge are part of a range of family, community, and health system factors that influence intention to breastfeed, which is in turn associated with improved breastfeeding practice [26]. Similarly, the association between EBF and not expressing breastmilk for bottle feeding after discharge is consistent with findings of a systematic review of seven trials including 1152 preterm infants [27], showing a negative impact of bottle feeding on breastfeeding practice. Bottle feeding is more likely to reduce lactation and bonding with the baby [25].

We did not find any associations between EBF at six months and factors that influence the provision of clinical care at the time of birth and may make breastfeeding more difficult, including caesarean section birth, timing of breastmilk expression or first feed, gestational age or weight, birth order, newborn respiratory support, and hospital readmission. Maternal medical problems were non-significantly associated with increased likelihood of EBF. Similarly, lack of early application of SSC or immediate and continuous KMC showed no significant association with reduced likelihood of EBF in this population. We believe that hospital policies and protocols targeted at improving staff practices and environments from birth to discharge, including rooming-in of mothers and babies in the NU, mitigated the negative effect of delayed SSC and KMC. Specifically, all mothers were supported to provide babies KMC and optimally feed breastmilk from the time of birth until discharge. In this environment, mothers with medical problems may have received additional support thereby making early and EBF more likely. Previous studies have suggested that lack of breastfeeding among women with high-risk obstetric conditions is associated with early separation, lack of counselling, and low intention to breastfeed, factors that can be overcome with adequate support [28].

Hospital and NU protocols in three areas were particularly important: immediate support for early and EBF and expression of breastmilk (including systems and practice changes around birth) implemented as part of EENC and early and sustained continuous KMC [3], support for appropriate feeding practices (human milk banking, lactation counselling, elimination of supplementary feeds, formula and use of bottles) [29,30], and an enabling environment in the NU and after discharge (breastfeeding friendly hospital policies, regular quality reviews, NU-based KMC, staff trained in breastfeeding counselling and support, and discharge planning including follow-up support after discharge) [29]. Keeping mothers and preterm infants together in the NU allows mothers to observe and respond to the infant's early feeding cues, breastfeed more often and have closer contact to increase bonding and engagement. Previous studies have indicated that allowing mothers to remain in the NU improves breastfeeding practice at three months and decreases length of hospital stays [31,32]. The result of this comprehensive approach to supporting non-separation and early breastfeeding was that all babies were EBF at discharge and one month; higher rates of EBF at discharge were previously found to be associated with longer durations of continued EBF [33].

Socio-economic variables (mother’s educational level, family income, work status and mothers age) and exposure to formula advertising were not significantly associated with EBF at six months, suggesting that barriers to feeding cut across all socio-economic and educational groups. Furthermore, antenatal breastfeeding counselling was not associated with EBF practice. Previous studies in Vietnam have shown that effective ante- and post-natal breastfeeding counselling and support have a positive effect on early and EBF [34]; however, health workers need to spend sufficient time and have skills to provide quality services for them to be effective [35]. We found a non-significant association between the likelihood of EBF at six-months and visiting public rather than private antenatal care clinics (OR = 3.2; 95% CI = 0.9-11.2). This is consistent with previous data suggesting that breastfeeding support is poorer and Code violations higher at private hospitals than in public hospitals [36]. Taken together, these data suggest that antenatal and postnatal breastfeeding counselling, education, and support needs strengthening at both public and private contacts. Incorporating breastfeeding counselling and support standards into health professional and facility licensing, licensing renewal, and accreditation systems should be a priority. More broadly, EBF is influenced by psychosocial, demographic, and environmental factors, with psychosocial factors such as self‐efficacy, intention to breastfeed, attitudes toward breastfeeding, and social support being associated with the duration of EBF [37]. Modifying perceptions and practices requires behaviour change and communication interventions delivered at health facilities, homes, communities, and through mass media. One-on-one communication can improve EBF practices and should be a part of this approach. Further, combined interventions such as interpersonal communication and mass media at different levels, including home, community and health facilities, have been demonstrated to improve EBF outcomes in Vietnam and elsewhere [38].

Despite relatively high EBF rates, immediate and continuing KMC were not applied for all preterm infants in the study population. Early separation of mothers and babies continues to occur, delaying or limiting feeding interventions. Further observation of current practices and workflow are needed to identify and address the underlying reasons for these practice gaps. Ward observations and focus group discussions with maternity and NU staff using study findings identified some on-going barriers to practice including: lack of coordination among obstetric, anaesthesiologic and neonatal staff, resulting in interrupted SSC or lack of breastfeeding support at certain periods after birth; maternal medical problems limiting their ability to provide immediate KMC; lack of neonatal staff to provide KMC monitoring of very preterm infants on respiratory support in delivery or operating rooms; and inadequate NU space for rooming-in at times of higher case-volume, limiting capacity for continuous KMC. Furthermore, ward observations and staff interviews suggested that breastfeeding counselling skills of staff can be highly variable, with staff in post-natal wards tending to be less effective because of more limited time availability and supervision, which is consistent with previous studies [39]. These facility factors, many related to set-up of care environments, organisation of workflow and quality of counselling practice can be targeted by on-going quality improvement actions.

This study provides further evidence that EBF can be improved for high-risk preterm infants using an integrated comprehensive quality improvement approach, based on non-separation of the mother and baby. This approach could be more widely adopted in Vietnam and other countries in the East Asia region, where most births are conducted in hospitals and management of preterm infants is an increasing challenge [8,40]. Behaviour change and communication interventions delivered at health facilities, homes, communities and through mass media remain important for facilitating continued EBF at home.

### Limitations

Since DNHWC is a tertiary referral hospital and patients referred for care may be at higher risk of complications, our study population may not be representative of all preterm infants in Vietnam or globally. However, its higher acuity is more likely to result in lower EBF rates compared to those born in facilities with less acuity. Sampling bias is possible if the 47 mothers of preterm infants (31.1%) lost to follow-up were different in key characteristics and outcomes from those included. The selection of only the first-born preterm infant in surviving multiple births may have introduced bias, if feeding practices and outcomes in first-borns were significantly different from subsequent births. Although mothers with multiple births can have more difficulty initiating and sustaining breastfeeding, we assumed that DNHWC policies to fully support maternal feeding practices for all mothers and babies regardless of birth number or order would prevent this from being a significant problem. We recognise that there are limited data available on this issue. A detailed examination of feeding patterns and outcomes for multiple preterm births is warranted to better investigate this question.

Mothers’ interviews may have been subject to recall bias. However, validity and reliability of recalled breastfeeding data are relatively high for survey data up to 24 months, and less likely to be a problem in the first six months of life, which was the case for breastfeeding practices in our study. Mothers may have been subject to reporting bias, i.e. wanting to please the interviewers by overreporting exclusive and under-reporting non-exclusive feeding and formula use. However, maternal responses were consistent across different social, economic, and educational domains, suggesting that this was not a significant problem. Interviewer training using standard questionnaires limited interviewer bias. Finally, we did not measure several factors potentially associated with EBF practice, including body mass, smoking, birth companion, availability of childcare or child support, and perinatal depression, which could be considered in subsequent studies to further identify and target breastfeeding barriers.

## CONCLUSIONS

We found that high risk preterm newborn infants born less than 34 weeks gestational age can be exclusively breastfed, given a strongly positive hospital environment. EBF rates were higher at discharge, one, three and six months than those reported previously in hospital-based, national or global studies, for both preterm and full-term newborn infants. To further improve breastfeeding outcomes, community, antenatal and postnatal care interventions should target improving client and family knowledge, skills and intention to breastfeed. Implementation of hospital protocols around birth need further strengthening to avoid feeding delays by ensuring early SSC and continuous KMC, improving counselling skills of staff, and organizing patient flow and space to facilitate mother baby contact. Given the relative simplicity of the interventions required to create a positive hospital breastfeeding environment, we believe that this approach is feasible for all hospitals.
